# Total torsion of three-dimensional lines of curvature

**DOI:** 10.1007/s10711-023-00833-8

**Published:** 2023-08-23

**Authors:** Matteo Raffaelli

**Affiliations:** Institute of Discrete Mathematics and Geometry, TU Wien, Wiedner Hauptstraße 8-10/104, 1040 Vienna, Austria

**Keywords:** Darboux curvatures, Parallel rotation, Three-dimensional curve, Total geodesic torsion, Primary 53A04, Secondary 53A07, 53C40

## Abstract

A curve $\gamma$ in a Riemannian manifold *M* is *three-dimensional* if its torsion (signed second curvature function) is well-defined and all higher-order curvatures vanish identically. In particular, when $\gamma$ lies on an oriented hypersurface *S* of *M*, we say that $\gamma$ is *well positioned* if the curve’s principal normal, its torsion vector, and the surface normal are everywhere coplanar. Suppose that $\gamma$ is three-dimensional and closed. We show that if $\gamma$ is a well-positioned line of curvature of *S*, then its total torsion is an integer multiple of $2\pi$; and that, conversely, if the total torsion of $\gamma$ is an integer multiple of $2\pi$, then there exists an oriented hypersurface of *M* in which $\gamma$ is a well-positioned line of curvature. Moreover, under the same assumptions, we prove that the total torsion of $\gamma$ vanishes when *S* is convex. This extends the classical total torsion theorem for spherical curves.

## Introduction and main result

In classical differential geometry, the *total torsion theorem* states that the total torsion of a closed spherical curve vanishes; see [3, 4, 10, 11] and [7, p. 170].

### Theorem 1.1

Let $I= [0, \ell ]$, and let $\gamma :I \rightarrow \mathbb {R}^{3}$ be a smooth regular curve. If $\gamma$ is closed and $\gamma (I)\in \mathbb {S}^{2}$, then

$$\begin{aligned} \int _{0}^{\ell } \tau \,dt = 0. \end{aligned}$$

Theorem 1.1 manifests the fact that “the torsion of a closed curve lying on a surface in $\mathbb {R}^{3}$ is somehow constrained by the geometry of [the] surface” [8, p. 111]; see, e.g., [1, 5, 6, 12] for further evidence of the same fact.

Closely related to Theorem 1.1 is the following result of Qin and Li.

### Theorem 1.2

[9]  Let *S* be a (smooth) oriented surface in $\mathbb {R}^{3}$. If $\gamma$ is a closed line of curvature of *S*, then the total torsion is an integer multiple of $2\pi$. Conversely, if the total torsion of a closed curve in $\mathbb {R}^{3}$ is an integer multiple of $2\pi$, then it can appear as a line of curvature of an oriented surface.

Theorem 1.1 and the first part of Theorem 1.2 have been generalized to three-dimensional orientable Riemannian manifolds of constant curvature $M_{c}^{3}$ [8]; see also [2, 15] for related results. In the present note we shall see that, under suitable assumptions, both theorems remain valid when $M^{3}_{c}$ is replaced by an arbitrary Riemannian manifold $M^{m}\,{\equiv }\,M$, provided one restricts the attention to *three-dimensional* curves; roughly speaking, a curve in *M* is three-dimensional if it has one curvature and one “torsion”, all other curvature functions being zero. As we explain below, in that case one should interpret “torsion” as a signed version of Spivak’s “second curvature function” [13, p. 22].

Let $\gamma$ be a unit-speed curve $I \rightarrow M$, let *N* be a unit normal vector field along $\gamma$, and let $\pi _{\mathcal {H}}$ be the orthogonal projection onto $\mathcal {H} = (\gamma ' \oplus N)^{\perp }$. We say that *N* is *torsion-defining* if there exists a smooth unit vector field *W*(*N*) along $\gamma$ that is everywhere parallel to $T_{g} = -\pi _{\mathcal {H}}D_{t}N$. If *N* is torsion-defining, then the function $\tau _{g} = \langle T_{g}, W(N)\rangle$ is called the *(first) geodesic torsion of* $\gamma$ *with respect to* *N*. In particular, if $D_{t}\gamma '$ is never zero, then the geodesic torsion of $\gamma$ with respect to the principal normal $P = D_{t}\gamma '/\kappa$ is called the *(first) torsion of* $\gamma$, and $\gamma$ is said to be a *Frenet curve*.

The logic behind our terminology is the following. In the same way a generic curve in $\mathbb {R}^{3}$ has one (unsigned) curvature plus one (signed) torsion, a generic curve in *M* may have one (unsigned) curvature plus $m-2$ (signed) torsions; cf. [13]. On the other hand, since we never deal with higher-order torsions, we typically speak of “torsion” as a shorthand for “first torsion”.

Now, to state our generalization of Theorems 1.1 and 1.2, let *S* be an oriented hypersurface of *M*, and let $N_S$ be its unit normal. A Frenet curve on *S* is said to be *well positioned* if $N_{S}$, *P*, and *W*(*P*) are everywhere coplanar.

### Theorem 1.3

Suppose that $\gamma$ is three-dimensional, i.e., that $\gamma$ is a Frenet curve such that *W*(*P*) is parallel in $\mathcal {H}(P)$; see Definition 6.1. If $\gamma$ is a well-positioned closed line of curvature of *S*, then the total torsion of $\gamma$ is an integer multiple of $2\pi$; in particular, the total torsion vanishes when *S* is convex, i.e., when the second fundamental form of *S* is positive definite. Conversely, if $\gamma$ is open, then there exists an orientable hypersurface in which $\gamma$ is a well-positioned line of curvature; if $\gamma$ is closed, then the same holds provided the total torsion of $\gamma$ is an integer multiple of $2\pi$.

Clearly, when $\dim M = 3$, every Frenet curve is three-dimensional, and every Frenet curve on *S* is well positioned. Specializing the theorem to that case, we may state the following result.

### Corollary 1.4

Suppose that $\dim M = 3$ and that $\gamma$ is a closed Frenet curve. If $\gamma$ is a line of curvature of *S*, then the total torsion of $\gamma$ is an integer multiple of $2\pi$; in particular, the total torsion vanishes when *S* is convex. Conversely, if the total torsion of $\gamma$ is an integer multiple of $2\pi$, then there exists an orientable surface in which $\gamma$ is a line of curvature.

### Remark 1.5

If $\dim M = 3$, then every regular curve with nonvanishing curvature is a Frenet curve.

We will obtain Theorem 1.3 as a corollary of a more general statement involving the geodesic torsion of $\gamma$ with respect to an arbitrary unit normal vector field *N* along $\gamma$, in which the assumption that $\gamma$ is three-dimensional is replaced by the condition that *N* is a *parallel rotation* of $N_{S}$.

Let *N* and *Z* be unit normal vector fields along $\gamma$. We say that *Z* is a *rotation of* *N* if there exists a continuous unit vector field $H(N, Z) \equiv H$ such that

1. $$\langle H, \gamma ' \rangle = \langle H, N \rangle = 0$$, i.e., $$H \in \Gamma (\mathcal {H})$$;
2. *H*, *N*, and *Z* are everywhere linearly dependent.

Clearly, if $N \wedge Z$ is nowhere zero, then the vector field *H* is defined up to a sign.

Now, suppose that *Z* is a rotation of *N*. Then we can write

$$\begin{aligned} Z \equiv N(\theta )= - \sin (\theta ) H + \cos (\theta ) N\ \end{aligned}$$

for some continuous function $\theta :I \rightarrow \mathbb {R}$.

### Definition 1.6

A rotation of *N* is said to be *parallel* if *H* is parallel with respect to the induced connection on $\mathcal {H}$, and *closed* if $\theta (\ell )-\theta (0) = 2n\pi$ for some $n \in \mathbb {Z}$.

### Remark 1.7

1. If $\dim M = 3$, then any unit normal vector field along $\gamma$ is a parallel rotation of *N*.
2. If $\gamma$ is closed, then so is any rotation of *N*.

### Theorem 1.8

If $\gamma$ is a line of curvature of *S*, then the total geodesic torsion of $\gamma$ with respect to any closed parallel rotation of $N_{S}$ is an integer multiple of $2\pi$. Conversely, suppose that *N* is torsion-defining and that *W*(*N*) is parallel in $\mathcal {H}$. If $\gamma$ is open, then there exists an orientable hypersurface of *M* in which $\gamma$ is a line of curvature; if $\gamma$ is closed, then the same holds provided the total geodesic torsion of $\gamma$ with respect to *N* is an integer multiple of $2\pi$.

### Remark 1.9

It follows from Sect. 4 that, if $\gamma$ is a line of curvature of *S* and *P* is a parallel rotation of $N_{S}$, then $\gamma$ is three-dimensional.

The remainder of the paper is organized a follows. In Sect. 2 we set up some notations. In Sect. 3 we generalize the well-known concepts of geodesic curvature, normal curvature, and geodesic torsion of a curve on a surface in $\mathbb {R}^{3}$ to a curve on a hypersurface of *M*; although, under reasonable assumptions, one may define $m-2$ geodesic curvatures and geodesic torsions, for the sake of simplicity we shall limit ourselves to first-order curvatures. In Sect. 4 we obtain formulas expressing the curvature vectors of $\gamma$ with respect to a rotation of *N* in terms of the rotation angle. Finally, in Sects. 5 and 6 we prove Theorems 1.8 and 1.3, respectively.

## Preliminaries

In this section we discuss some preliminaries.

Let *M* be an *m*-dimensional Riemannian manifold, let $\gamma$ be a smooth unit-speed curve $I \rightarrow M$, and let $TM |_{\gamma }$ be the ambient tangent bundle over $\gamma$. Recall that

$$\begin{aligned} TM|_{\gamma } = \bigsqcup _{t \in I} T_{\gamma (t)}M. \end{aligned}$$

We define a *distribution of rank* *r* *along* $\gamma$ to be a rank-*r* subbundle of $TM |_{\gamma }$.

Let $\mathcal {D}$ be a distribution of rank *r* along $\gamma$, and let $\mathcal {D}^{\perp }$ be the distribution of rank $m-r$ along $\gamma$ whose fiber at *t* is the orthogonal complement $\mathcal {D}_{t}^{\perp }$ of $\mathcal {D}_{t}$ in $T_{\gamma (t)}M$, so that $TM|_{\gamma }$ splits as

$$\begin{aligned} TM|_{\gamma } = \mathcal {D} \oplus \mathcal {D}^{\perp }; \end{aligned}$$

accordingly, we write

$$\begin{aligned} X = X^{v} + X^{h} \end{aligned}$$

for any vector field *X* along $\gamma$.

In this setting, the *tangential projection* is the map $\pi _{\mathcal {D}} :\Gamma (TM|_{\gamma }) \rightarrow \Gamma (\mathcal {D})$ given by

$$\begin{aligned} X \mapsto X^{v}. \end{aligned}$$

Likewise, the *normal projection* is the map $\pi ^{\perp }_{\mathcal {D}} :\Gamma (TM|_{\gamma }) \rightarrow \Gamma (\mathcal {D}^{\perp })$ sending each *X* to the corresponding $X^{h}$.

## Darboux curvatures and curvature vectors

The purpose of this section is to extend the classical notions of geodesic curvature, normal curvature, and geodesic torsion of a curve on a surface in $\mathbb {R}^{3}$ to a curve on a hypersurface of *M*.

Let $\gamma$ be a (smooth) unit-speed curve $I \rightarrow M$, let *N* be a unit normal vector field along $\gamma$, and let $\mathcal {H}(N) \equiv \mathcal {H}$ be the distribution of rank $m-2$ along $\gamma$ whose fiber at *t* is the orthogonal complement of $E(t) = \gamma '(t)$ and *N*(*t*) in $T_{\gamma (t)}M$. Denoting by $D_{t}$ the covariant derivative along $\gamma$, we define

- the *(first) geodesic curvature vector* $K_{g}$ *of* $\gamma$ *with respect to* *N* by
  $$\begin{aligned} K_{g} = \pi _{\mathcal {H}} D_{t}E; \end{aligned}$$
- the *normal curvature vector* $K_{n}$ *of* $\gamma$ *with respect to* *N* by
  $$\begin{aligned} K_{n} = \pi _{\mathcal {N}} D_{t}E, \end{aligned}$$
  where $$\mathcal {N} = {{\,\textrm{span}\,}}N$$;
- the *(first) geodesic torsion vector* $T_{g}$ *of* $\gamma$ *with respect to* *N* by
  $$\begin{aligned} T_{g} = -\pi _{\mathcal {H}} D_{t}N. \end{aligned}$$

To express these vector fields in coordinates, let $(H_{1}, \dotsc , H_{m-2})$ be a smooth orthonormal frame for $\mathcal {H}$. Then there are functions $\kappa _{g}^{1}, \dotsc , \kappa _{g}^{m-2}$, $\kappa _{n}$, and $\tau _{g}^{1}, \dotsc , \tau _{g}^{m-2}$ such that

$$\begin{aligned} K_{g}&= \kappa _{g}^{1}H_{1} + \cdots + \kappa _{g}^{m-2} H_{m-2},\\ K_{n}&= \kappa _{n} N,\\ T_{g}&= \tau _{g}^{1}H_{1} + \cdots + \tau _{g}^{m-2} H_{m-2}. \end{aligned}$$

Note that, since $(E, H_{1}, \dotsc , H_{m-2}, N)$ is orthonormal, the following equations hold for all $j =1, \dotsc , m-2$:

$$\begin{aligned}&D_{t}E = \kappa _{g}^{1}H_{1} + \cdots + \kappa _{g}^{m-2}H_{m-2} + \kappa _{n}N, \\&D_{t}H_{j} = -\kappa _{g}^{j}E + \tau _{g}^{j}N + \pi _{\mathcal {H}} D_{t}H_{j}, \\&D_{t}N = -\kappa _{n}E - \tau _{g}^{1}H_{1} - \cdots - \tau _{g}^{m-2} H_{m-2}. \end{aligned}$$

The curvature vectors allow us to define corresponding curvature *functions*. In one case, the definition is trivial: the function $\kappa _{n} = \langle D_{t}E, N \rangle$ is called the *normal curvature of* $\gamma$ *with respect to* *N*. For the remaining two cases, we proceed as follows.

We say that *N* is *curvature-defining* if there exists a smooth unit vector field $V(N) \equiv V$ along $\gamma$ that is everywhere parallel to $K_{g}$. If *N* is curvature-defining, then the function $\kappa _{g} = \langle K_{g}, V \rangle$ is called the *(first) geodesic curvature of* $\gamma$ *with respect to* *N*.

Similarly, we say that *N* is *torsion-defining* if there exists a smooth unit vector field $W(N) \equiv W$ along $\gamma$ that is everywhere parallel to $T_{g}$. If *N* is torsion-defining, then the function $\tau _{g} = \langle T_{g}, W \rangle$ is called the *(first) geodesic torsion of* $\gamma$ *with respect to* *N*.

It is clear that both $\kappa _{g}$ and $\tau _{g}$ are defined up to a sign.

Armed with the notion of geodesic torsion, we may now define torsion. Suppose that the curvature $\kappa = \Vert D_{t}E \Vert$ of $\gamma$ is nowhere zero, so that the *principal normal* $P = D_{t}E/\kappa$ is well-defined. The geodesic torsion vector of $\gamma$ with respect to *P* is called the *(first) torsion vector of* $\gamma$. In particular, if *P* is torsion-defining, then the geodesic torsion of $\gamma$ with respect to *P* is called the *(first) torsion of* $\gamma$.

### Remark 3.1

If *P* is well-defined, then the normal curvature of $\gamma$ with respect to *P* coincides with the curvature of $\gamma$, while the geodesic curvature with respect to *P* vanishes.

To see that our curvature functions naturally extend the classical Darboux curvatures, consider an oriented hypersurface *S* of *M*, and let $N_{S}$ be its unit normal. If $\gamma$ is a curve on *S*, then the geodesic (resp., normal) curvature vector of $\gamma$ (with respect to $N_{S}$) is the projection onto *TS* (resp., *NS*) of the ambient acceleration $D_{t}E$ of $\gamma$; and if $\gamma$ is not a geodesic of *M*, then the geodesic torsion vector of $\gamma$ at $\gamma (t)$ is nothing but the torsion vector of the *S*-geodesic passing from $\gamma (t)$ with tangent vector $\gamma '(t)$ [14, p. 193].

Yet another indication of the naturality of our definition of geodesic torsion is provided by the following lemma, which will play a key role in the proof of Theorem 1.8.

### Lemma 3.2

A curve on *S* is a line of curvature if and only if its geodesic torsion vector with respect to $N_{S}$ vanishes.

### Remark 3.3

Under suitable assumptions, one may define $m-2$ geodesic curvature and (geodesic) torsion functions. For instance, the second geodesic torsion is defined as follows. Let $\mathcal {H}_{2} = (T \oplus N \oplus T_{g})^{\perp }$, let $\pi _{\mathcal {H}_{2}}$ be the orthogonal projection onto $\mathcal {H}_{2}$, and let

$$\begin{aligned} T_{g,2} = -\pi _{\mathcal {H}_{2}} D_{t}T_{g}. \end{aligned}$$

If $T_{g}$ is itself torsion-defining, i.e., there exists a smooth unit vector field $W_{2}$ along $\gamma$ that is everywhere parallel to $T_{g,2}$, then the function $\tau _{g,2} = \langle T_{g,2}, W_{2} \rangle$ is called the *second geodesic torsion of* $\gamma$ *with respect to* *N*. Higher-order geodesic torsions are defined similarly.

## Rotating the normal

Suppose that the normal vector *N* along $\gamma$ rotates about the curve’s tangent. Then how do the curvature vectors change? The purpose of this section is to answer such question.

Let *Z* be a rotation of *N*. Then, by definition, there exists a unit normal vector field $H(N,Z) \equiv H \in \Gamma (\mathcal {H})$ along $\gamma$ such that *N*, *Z*, and *H* are everywhere linearly dependent; besides, there is a continuous function $\theta :I \rightarrow \mathbb {R}$ such that

$$\begin{aligned} Z = -\sin (\theta )H + \cos (\theta )N. \end{aligned}$$

Denoting *Z* by $N(\theta )$, we call the function $\theta$ the *rotation angle of* $N(\theta )$ *with respect to* *H*.

Now, let $(H_{1}, \dotsc , H_{m-2})$ be a smooth orthonormal frame for $\mathcal {H} = (E \oplus N)^{\perp }$, with $H_{1} = H$. It follows that

$$\begin{aligned} N(\theta ) = -\sin (\theta ) H_{1} + \cos (\theta ) N, \end{aligned}$$

while the vector fields

$$\begin{aligned} H_{1}(\theta )&= \cos (\theta )H_{1} + \sin (\theta ) N,\\ H_{2}(\theta )&= H_{2},\\&\quad {\vdots } \\ H_{m-2}(\theta )&= H_{m-2} \end{aligned}$$

span $$\mathcal {H}(N(\theta )) = (E \oplus N(\theta ))^{\perp }$$.

### Lemma 4.1

The curvature vectors of $\gamma$ with respect to $N(\theta )$ are given by

$$\begin{aligned} K_{g}(\theta )&= \left( \kappa _{g}^{1} c + \kappa _{n} s \right) H_{1}(\theta ) + \kappa _{g}^{2} H_{2}(\theta ) + \cdots + \kappa _{g}^{m-2} H_{m-2}(\theta ),\\ K_{n}(\theta )&= \left( -\kappa _{g}^{1} s + \kappa _{n} c \right) N(\theta ),\\ T_{g}(\theta )&= \left( \theta ' + \tau _{g}^{1} \right) H_{1}(\theta ) + \left( \tau _{g}^{2} c - \mu _{2} s \right) H_{2}(\theta ) + \cdots + \left( \tau _{g}^{m-2} c - \mu _{m-2} s \right) H_{m-2}(\theta ), \end{aligned}$$

where $\mu _{j} = \langle D_{t} H_{j}, H_{1} \rangle$, and where *c* and *s* are shorthands for $\cos (\theta )$ and $\sin (\theta )$, respectively.

## Proof of Theorem 1.8

Here we prove our most general result, Theorem 1.8 in the introduction.

To begin with, suppose that $\gamma$ is a line of curvature of *S* and that $N_{S}(\theta )$ is a parallel rotation of $N_{S}$. Then the geodesic torsion of $\gamma$ with respect to $N_{S}$ vanishes and the vector field $H(N_{S}, N_{S}(\theta ))$ is parallel in $\mathcal {H}$.

Let $(H_{1}, \dotsc , H_{m-2})$ be a smooth orthonormal frame for $(E \oplus N_{S})^{\perp }$ such that $H_{1} = H$. Applying Lemma 4.1, we deduce that $T_{g}(\theta ) = \theta ' H_{1}(\theta )$, which implies that $N_{S}(\theta )$ is torsion-defining and that $\theta ' = \pm \tau _{g}(\theta )$.

Since

$$\begin{aligned} \int _{0}^{\ell } \theta ' \, dt = \theta (\ell ) - \theta (0), \end{aligned}$$

it follows that, when $N_{S}(\theta )$ is a closed rotation of $N_{S}$,

$$\begin{aligned} \int _{0}^{\ell } \tau _{g}(\theta ) = 2n\pi \quad \hbox { for some}\ n \in \mathbb {Z}, \end{aligned}$$

as desired.

Conversely, given any (torsion-defining) unit normal vector field *N* along $\gamma$, suppose that $W(N) \equiv W$ is parallel in $\mathcal {H}$. Choose an orthonormal frame $(H_{1}, \dotsc , H_{m-2})$ for $\mathcal {H}$, with $H_{1} = W$, and let

$$\begin{aligned} N(\theta )&= -\sin (\theta )H_{1} + \cos (\theta )N,\\ H_{1}(\theta )&= \cos (\theta )H_{1} + \sin (\theta )N, \end{aligned}$$

where

1 $$\begin{aligned} \theta (t) = -\int _{0}^{t} \tau _{g}(s) \, ds. \end{aligned}$$

(Note that $N(\theta )$ is a parallel rotation of *N*.)

Define a map $\sigma :[0, \ell ] \times \mathbb {R}^{m-1} \rightarrow M$ by

$$\begin{aligned} \sigma (t, u) = \exp _{\gamma (t)}\left( u^{1}H_{1}(\theta )(t) + u^{2}H_{2}(t) + \cdots + u^{m-2} H_{m-2}(t)\right) . \end{aligned}$$

It is clear that $\sigma$ is a smooth immersion in a neighborhood of $[0, \ell ] \times \{0 \}$; besides, its image is normal to $N(\theta )$ along $\gamma$.

It remains to show that $\gamma$ is a line of curvature of $\sigma$, i.e., that the geodesic torsion $\tau _{g}(\theta )$ of $\gamma$ with respect to $N(\theta )$ vanishes. Differentiating (1), we have

$$\begin{aligned} \theta ' = -\tau _{g}^{1}, \end{aligned}$$

which implies $\tau _{g}^{1}(\theta ) = 0$, as desired. Since $\tau _{g}^{2} = \cdots = \tau _{g}^{m-1} = 0$ and $H_{1}$ is parallel in $\mathcal {H}$, we conclude that $\tau _{g}(\theta ) = 0$ by Lemma 4.1.

## Three-dimensional curves

Let $\gamma :I \rightarrow M$ be a Frenet curve, let $H_{1} = W(P)$, and let $(H_{2}, \dotsc , H_{m-2})$ be a parallel frame for the orthogonal complement of $H_{1}$ in $\mathcal {H}(P)$.

### Definition 6.1

We say that $\gamma$ is *three-dimensional* if the following equations hold:

$$\begin{aligned} D_{t}E&= \kappa P,\\ D_{t}H_{1}&= \tau P,\\ D_{t}H_{2}&= \cdots = D_{t}H_{m-2} =0,\\ D_{t}P&= -\kappa E -\tau H_{1}. \end{aligned}$$

It is clear that $\gamma$ is three-dimensional if and only if *W*(*P*) is parallel in $\mathcal {H}(P)$.

The purpose of this section is to prove Theorem 1.3 in the introduction.

### Proof of Theorem 1.3

Suppose that *P* is a parallel rotation of $N_{S}$, and let $\theta$ be the rotation angle of *P* with respect to *W*(*P*). We know from the proof of Theorem 1.8 that if $\gamma$ is a line of curvature and *P* is a *closed* rotation, then

2 $$\begin{aligned} \pm \int _{0}^{\ell } \tau \, dt = \theta (\ell ) - \theta (0) = 2n\pi \quad \hbox { for some}\ n \in \mathbb {Z}. \end{aligned}$$

On the other hand, applying Lemma 4.1, we observe that the normal curvature of $\gamma$ with respect to $N_{S}$ is related to the curvature $\kappa$ by the relation

$$\begin{aligned} \kappa _{n} = \kappa (\theta ) = \kappa \cos (\theta ). \end{aligned}$$

Suppose that *M* is convex, so that $\kappa _{n} > 0$. Since $\kappa >0$, we have $\cos (\theta ) > 0$, from which we conclude that

$$\begin{aligned} \theta (\ell ) - \theta (0) \in (-\pi , \pi ). \end{aligned}$$

Together with (2), this implies $n =0$, as desired. $\square$
